# Adipocytes from lipedema adipose tissue show lipoma-associated nuclear atypia

**DOI:** 10.3389/fcell.2026.1804040

**Published:** 2026-05-01

**Authors:** M. Steiner, M. Rinnerthaler, A. Mueller, E. Russe, A.-Th. Lipp, H. Bauer

**Affiliations:** 1 Department of Biosciences and Medical Biology, Paris Lodron University of Salzburg, Salzburg, Austria; 2 Department of Plastic, Reconstructive and Aesthetic Surgery, Hospital of the Brothers of St. John of God, Paracelsus Medical University, Salzburg, Austria; 3 PANTEA Private Practice for Plastic & Aesthetic Surgery and Lipedema Surgery, Munich, Germany

**Keywords:** adipocyte nuclear atypia, lipedema, lipoma, lipomatosis, lochkerne

## Abstract

**Introduction:**

Lipedema, a painful disease that almost exclusively affects women, leads to an excessive accumulation of subcutaneous adipose tissue, primarily in the extremities. Morphologically, it is characterized by hyperplasia and hypertrophy of adipocytes as well as by inflammation-associated cells and fibrosis. Limited knowledge exists regarding the background of adipocyte pathology. In the present study, we aimed to identify morphological alterations of lipedema adipocytes, which could cause functional implications in lipedema adipose tissue.

**Methods:**

Approximately 3,000 adipocytes from nine lipedema and five control adipose tissue samples, originating from non-obese donors, were analyzed. The ratio of atypical nuclei (*Lochkerne*) in relation to the total amount of nuclei was assessed and compared between lipedema and non-lipedema samples.

**Results:**

Lipedema adipose tissue exhibits a significantly higher proportion of *Lochkerne* compared to controls (p = 0.001). While 24% of adipocyte nuclei presented as *Lochkerne* in lipedema samples, only 3% were identifiable in controls. We further show that the process of *Lochkern*-formation involves the nuclear indentation by small lipid droplets and their subsequent transmigration through the nucleus towards the central lipid content.

**Conclusion:**

The significantly increased occurrence of lipoma-associated *Lochkerne* in lipedema adipose tissue compared to controls reveals that, from a morphological point of view, lipedema is a form of lipomatosis.

## Introduction

Although lipomas are a characteristic marker for several well-described lipomatoses, they have never been of diagnostic value for lipedema. Information concerning the incidence of lipomas in lipedema is particularly limited. While some case studies reported the occurrence of single or multiple lipomas in lipedema patients (Shin et al., 2011; Pascucci and Lynch, 2010), the presence of lipomas appears to be the exception rather than the rule in lipedema pathology. The obligatory occurrence of lipomas in Dercum´s disease (DD) has even been considered one of the distinguishing features between DD and lipedema (Beltran and Herbst, 2017).

Interesting results from a genome-wide association study revealed multiple genomic loci, suggestively associated with lipedema (Grigoriadis et al., 2022). The authors reported on single nucleotide polymorphisms located in a regulatory region of LHFPL6, a member of the lipoma HMGIC fusion partner (LHFP) gene family. LHFPL6 is a tetraspan protein, supposed to play a role in translocation-associated lipoma formation (Petit et al., 1999). The authors further suggest that lipedema patients carrying the critical variants upstream of LHFPL6 appear to be significantly more affected by a familiar form of lipedema.

While most lipomas are located above or below the superficial fascia, a minor portion can be found underneath the deep fascia, presenting as intramuscular (McTighe and Chernev, 2014) or intermuscular lipomas (Nishida et al., 2007). Rarely, lipomas develop in the intestines, lungs, heart, synovial tissue, tendon sheaths, joints, near or on bones, and even in nerve tissue (Gupta et al., 2016; Bancroft et al., 2006).

It is important to emphasize that lipomatosis can also present as a diffuse mass of excessive adipose tissue without clearly defined encapsulated lipomas. For instance, Madelung’s disease is characterized by the accumulation of non-encapsulated fat deposits (Chen et al., 2018; Szewc et al., 2018). Similarly, diffuse abdominal infiltrating lipomatosis (Ure et al., 2016) or congenital infiltrating lipomatosis of the face (Li et al., 2018) are two rare pathological conditions that manifest as non-encapsulated overgrowth of adipose tissue.

As far as lipedema (lipalgia, *lipohyperplasia dolorosa*) is concerned, it is sometimes classified as lipomatosis (Dupuis et al., 2024). In fact, lipedema usually manifests as a local accumulation of adipose tissue unrelated to obesity, with varying degrees of fibrosis (Kruppa et al., 2020; Ernst et al., 2022; Herbst et al., 2015; Poojari et al., 2022; Herbst et al., 2000). However, a more detailed examination of all medical evidence available so far shows that there is still no conclusive histological data that proves a lipoma-like character of lipedema adipose tissue.

Using transmission electron microscopy (TEM) and light microscopy we have set out to characterize lipedema adipose tissue at the fine structural and ultrastructural level focusing on potential lipoma-like structural characteristics.

Here we show for the first time that adipocytes of the subcutaneous lipedema adipose tissue exhibit nuclear atypia, which is normally attributed to lipogenic tumors and is scarcely found in normal adipocytes or other tissues. The identification of these so-called *Lochkerne* in lipedema adipocytes and the demonstration of their gradual formation in lipedema adipose tissue support the assumption that lipedema can be considered a genuine form of lipomatosis from a morphological perspective.

## Materials and methods

### Patients and sample taking

Biopsied subcutaneous adipose tissue from patients diagnosed with lipedema (N = 9) with a mean BMI of 26.1 kg/m2 and a mean age at surgery of 33.8 years as well as from healthy control donors (N = 5) with a mean BMI 22.8 kg/m2 with a mean age of 44.4 years was analyzed. Samples were collected from the extremities (upper arms and lateral thighs) of lipedema and non-lipedema donors. None of the patients exhibited pronounced fibrosis. All samples were taken in the course of first or second liposuction. Before infiltration of tumescent solution, an approximately 3 mm^3^ tissue specimen was excised from the subfascial layer (“deep adipose tissue”) using surgical scissors. Samples were immediately transferred to various fixatives as described below.

Informed consent was acquired from all patients prior surgery. All experimental protocols were approved by the Ethics Committee (Ethics votum 1142/2020) of Salzburg, Austria.

### Electron and light microscopy on epoxide resin sections

Biopsied tissue was stabilized by immersion in primary Karnovky´s fixative (5% glutaraldehyde/4% formaldehyde in 0.1M cacodylate buffer, pH 7.3) for 20 min before cutting into small blocks (<1 mm^3^) and continuing fixation for 6–8 h at room temperature. Extended fixation was performed for at least 24 h at 4 °C.

Secondary fixation was carried out using 1% osmium tetroxide in 0.1 M cacodylate buffer pH 7.3 for at least 12 h at 4 °C. After each fixation step the samples were washed thoroughly in 0.1 M cacodylate buffer. For embedding in epoxy resin (Agar 100, Agar Scientific, UK) the samples were dehydrated in ethanol and propylene oxide, infiltrated with graded mixtures of propylene oxide and resin followed by incubation in pure resin at 4 °C overnight. Tissue blocks were embedded in fresh resin in silicone molds and polymerized for 24 h at 60°C.

Semithin (0.5 μm) and ultrathin sections (0.07 μm) were cut using an Ultracut S ultramicrotome (Leica, Germany). For light microscopic analysis, semithin sections were stained with azurII/methylene blue (Richardson’s stain) and inspected with a Nikon eclipse 800 light microscope equipped with a Nikon Ds-Ri camera. Ultrathin sections were further prepared for electron microscopy, by double contrasting with uranyl acetate and lead citrate. Analysis and image recordings were carried out on a Zeiss transmission electron EM 900 equipped with a slow-scan CCD 2K wide-angle dual speed camera (TRS, Moorenweis, Germany).

### Light microscopy on paraffin sections

Biopsies (≈5 mm^3^) were fixed with 4% PFA in PBS for at least 24 h, stored in 0.2% PFA, washed with PBS and embedded by an automated procedure in a Leica tissue processor.

Paraffin sections (4 µm) were cut on a Leica HistoCore Autocut microtome (Leica, Germany). Deparaffinized sections were stained with hematoxylin and eosin. Micrographs were taken with an Olympus BX bright field Olympus OMD E−1 Mark II digital camera, using OM capture software (OM Digital Solutions).

### Statistical analysis

For statistical analysis, the percentage of Lochkerne was first calculated for each patient sample and subsequently compared between groups using a two-sided independent samples t-test.

## Results

**FIGURE 1:**
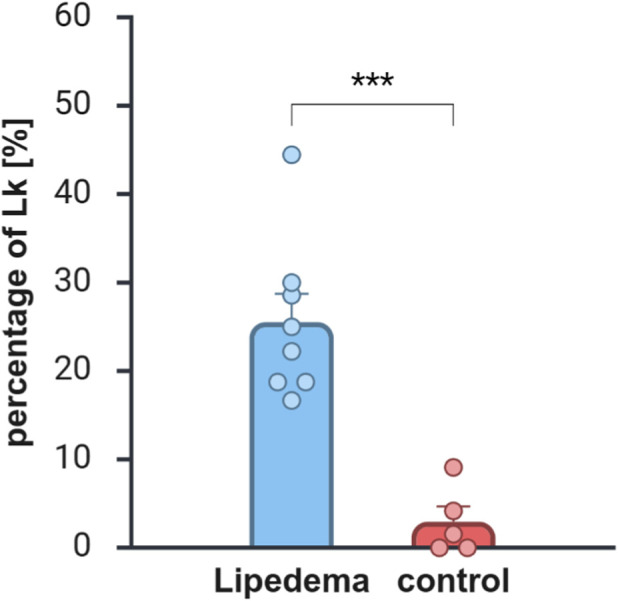
Comparison of *Lochkern* frequency in lipedema and control subcutaneous adipose tissue. Lipedema adipose tissue (N = 9; n = 154); control tissue (N = 5; n = 121). ***: p < 0.001 (Created in BioRender. Rinnerthaler, M. (2026) https://BioRender.com/vj27hj4).

We have shown that the percentage of nuclear indentations (*Lochkerne)* in subcutaneous lipedema adipose tissue is significantly higher than in control tissue (Figure 1). In summary, more than 3000 adipocyte cross-sections from normal and lipedema tissue samples were analyzed, wherein 275 nuclei were clearly detectable. 121 nuclei from control sections and 154 nuclei from lipedema sections were evaluated, respectively. In both groups, a comparably small number of unidentifyable nuclei was found and excluded from analysis.

**FIGURE 2:**
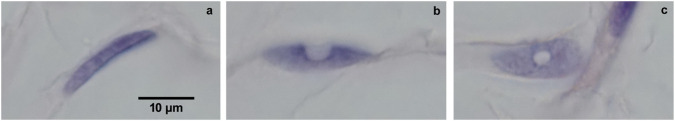
Histo-morphological appearance of adipocyte nuclei, shown in paraffin sections stained with hematoxylin/eosin. Lipids are absent due to the paraffin embedding process. **(a)** Sagittal plane of a nucleus without indentation. **(b)** Adipocyte nucleus showing indentation by a lipid droplet appearing as *“Kerbenkern”*. **(c)** Adipocyte nucleus cut in a spherical plane, showing a centered “hole” appearing as vacuolated nucleus (ring nucleus).

In the paraffin sections several forms of indentations in adipocyte nuclei are shown (Figure 2). Lochkerne, notched nuclei, as well as ring nuclei originally described in earlier publications could be observed (see discussion). Since this classification later fell out of use, we exclusively use the term *Lochkerne* for all forms of nuclear indentation in this work.

With regard to the cutting planes of paraffin sections, spherical and sagittal planes were equally frequent.

**FIGURE 3:**
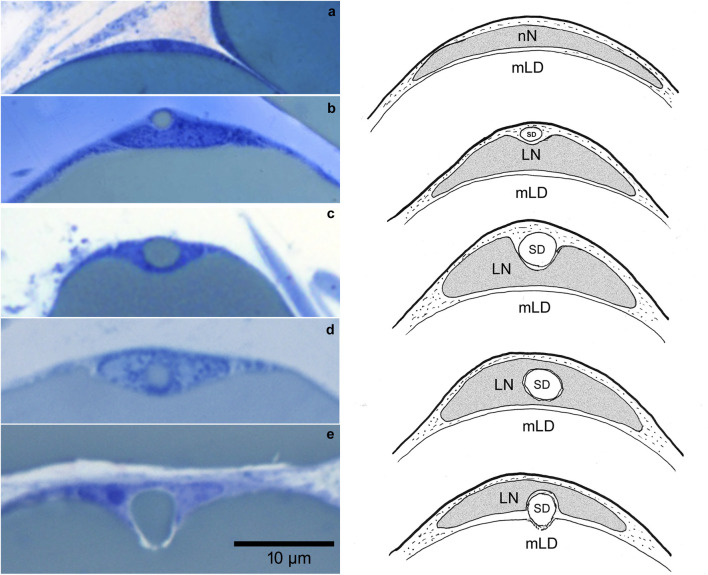
**(a)**, **(b)** Gradual formation of a *Lochkern* as depicted in semithin sections. The single steps of nuclear indentation in a mature lipedema adipocyte (left panel) with corresponding schematic drawings (right panel) is shown. **(a)** Semithin section (sagittal plane) of a normal nucleus (nN) stained with Richardson´s dye solution without indentation in non-lipedema control adipose tissue. **(b)** A satellite droplet (SD) approaches and indents the nucleus from a transposition. **(c)** A SD is enveloped by the nucleus. Thereby the nucleus is strongly deformed. **(d)** The nucleus completely surrounds the SD, while the droplet passes through the nucleus. **(e)** The lipid droplet leaves the nucleus through an opening of the nuclear membrane. The phospholipid membrane appears dissolved (white halo). Schematic drawing, summarizing the process of Lochkern formation (attachment, indentation, transmigration and exit of the SD to the main lipid droplet (mLD) corresponding to the semithin sections.

Due to the preservation of lipids, we were able to trace the genesis of individual droplets, in the following referred to as *satellite droplets* (SD*)*, located in a transposition in respect to the nucleus and the main droplet. Semithin sections (Figures 3a–e) and a schematic representation show the step-by-step process of *Lochkern* formation. Thereby, a satellite droplet approaches and indents the nucleus. After transmigration through the nucleus, the SD attaches to and fuses with the main droplet.

Due to the particularly small thickness of the epoxy resin semithin (0.5 µm) and ultrathin (70 nm) sections compared to the enormous size of the adipocytes (diameter 130 µm and more), predominantly sagittal sections were visible. In contrast to paraffin sections, nuclei in semithin and ultrathin sections were nearly always clearly identifyable. Since the difference in the frequency of *Lochkerne* compared to normal nuclei is evident, light microscopic analysis of semithin sections is sufficient for clarifying *Lochkern* occurrence.

**FIGURE 4:**
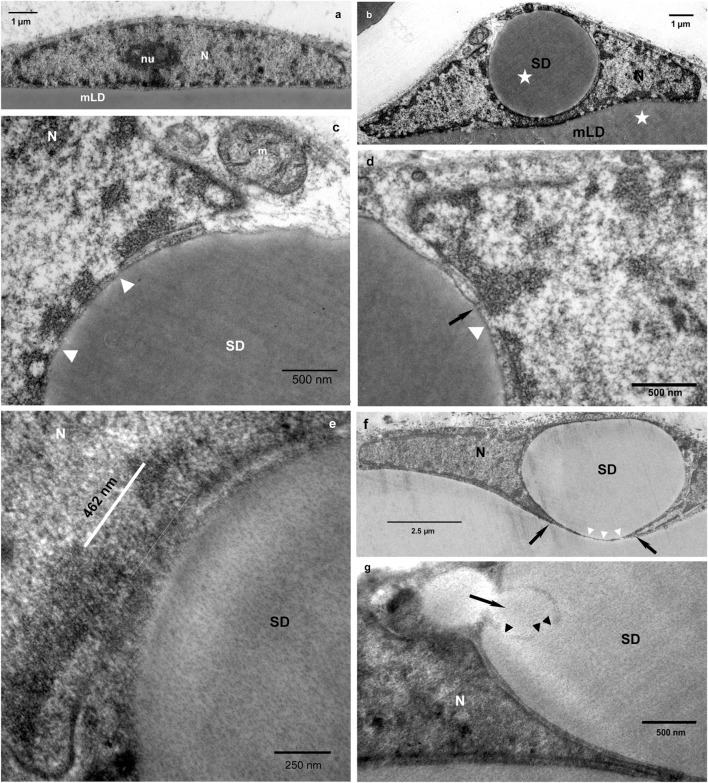
**(a)** Adipocyte nucleus (N) with a pronounced nucleolus (nu) without indentation in non-lipedema control tissue (sagittal plane). The nucleus appears flat, adapting its form to the curvature of the mature adipocyte and is closely attached to the main lipid droplet (mLD). **(b)** *Lochkern* in a lipedema adipocyte (sagittal plane). The bulging of the nucleus, due to the indentation, is seen. Electron optical analysis clearly indicates that the content of the satellite droplet (SD) and the mLD is identical (asterisks). **(c)** Ultrathin sections showing the contact zone between the nucleus and the SD at the nuclear pore (arrowheads). A mitochondrion is seen within the adipocyte cytoplasm (m). **(d)** A channel-like stucture connects the SD with the adjacent heterochromatin (arrow) and the nuclear pore contact (arrowhead). **(e)** High magnification of a strongly extended contact region [462 nm, measured by ImageJ] between a satellite droplet and the nuclear membrane. **(f)** A Lochkern showing a fenestration for passage of the satellite droplet to the main droplet. Black arrows indicate the opening within the nuclear membrane, white arrowheads point to the dissolution of the phosholipid membrane. **(g)** A lateral small lipid droplet is fusing (black arrow) with the large satellite droplet. This fusion process (arrowheads) is identical with fusion of normally generated LDs during lipogenesis.

At the ultrastructural level, the typical image of a normal nucleus with a prominent nucleolus was frequently observed (Figure 4a). Figure 4b shows *Lochkern* formation, comprising growth and enlargement of a SD which deeply indents the nucleus. Various details which were found to be characteristic for the contact zone between the nucleus and the SD are shown (Figures 4c,d). The open contact region (lacking the nuclear membrane) between nucleus and SD can exeed the size of the nulear pore and even that of the nuclear pore complex (Figure 4e). TEM analysis confirms our data from semithin sections, showing the transmigration of the satellite droplet through the nucleus (Figure 4f). After transmigration, the SD leaves the nucleus and subsequently merges with the main droplet, which is marked by the diappearance of the phospholipid membranes (pale surrounding) (Figure 4g).

## Discussion

Results from our histological study demonstrate that lipedema adipocytes exhibit structural characteristics, termed *Lochkerne,* which are usually present in lipogenic lesions (Stacy et al., 2014; Taweevisit and Thorner, 2024; Resnik and Kutzner, 2004). Originally, the German term *Lochkern* (meaning: “nucleus with a hole”) referred to an actual hole in the nucleus of adipocytes, whereas in later publications it was increasingly recognized that the characteristic *Lochkern*-appearance could be the result of nuclear invagination by the cytoplasm, creating a pocket in the nuclear membrane. Such invaginations can lead to different nuclear shapes, which have been designated as ring nuclei (*Ringkerne*), notch nuclei (*Kerbenkerne*), and hole nuclei (*Lochkerne*) (Resnik and Kutzner, 2004; Bavle, 2016; Winckler, 1960).

It must be emphasized that the presence of *Lochkerne* in adipocytes is not a pathologic finding, provided that such nuclei are not additionally hyperchromatic or pleomorphic. This is an important feature that distinguishes them from the nuclei of lipoblasts found in liposarcomas (Hisaoka, 2014). Our investigations revealed no evidence of lipoblasts in either the lipedema samples or the control samples. Interestingly, quantitative assessment revealed that the frequency of *Lochkerne* was unrelated to BMI and age in our samples.

Importantly, our results do not support the notion that the appearance of *Lochkerne* in adipocytes could be the result of *de novo* intranuclear droplet formation, which may occasionally be observed in various tissues (Fujimoto, 2022). We have clearly shown that *Lochkern* formation starts with small single droplets approaching and indenting the nucleus from a *transposition*, i.e., from the narrow cytoplasmic space between the nucleus and the plasma membrane of the adipocyte arising by its endoplasmic reticulum. Very scarcely, more than one droplet could be found to depress the nucleus of lipedema adipocytes.

From our observations we deduced a hypothetical temporal sequence of single steps, leading to the uptake and translocation of the satellite droplets into and through the indented nucleus. The final fusion of a satellite droplet with the main lipid content was seldomly detectable. The stages described above were found at varying frequencies in our histological sections, which might be due to their different duration.

Nuclear indentation by lipid droplets in adipogenically induced osteosarcoma cells has been reported by Ivanovska et al. (2023), showing remarkable rigidity of the indenting lipid droplets. Similarly, Verstraeten et al. demonstrated the indentation of adipocyte nuclei in an *in vitro* model of adipogenic induction and in electron micrographs of biopsied skin tissue (Verstraeten et al., 2011).

Interestingly, the frequency of *Lochkerne* found in lipedema adipose tissue corresponds to that of indented nuclei in the artificially induced lipogenic *in vitro* models described above. Although our morphological data do not allow a conclusion concerning the trigger of the nuclear indentation in lipedema adipocytes it may be speculated that this phenomenon is the result of overactive lipogenesis.

The consequence of nuclear indenture is still unknown. It is conceivable that any change in nuclear shape affects nuclear biology and impacts gene expression due to chromatin reorganization (Venturini et al., 2020; Dupouy et al., 2024).

Severe DNA damage and loss of key DNA repair mechanisms following nuclear rupture of indented adipocyte nuclei were reported (Ivanovska et al., 2023). Although our electron optical images do not depict any signs of nuclear rupture it cannot be ruled out that the strong deformation of the nucleus during the indentation and transmigration of the SD is sufficient to trigger chromatin rearrangement and associated consequences for gene expression. However, as the present study is based solely on morphological and ultrastructural observations, such functional implications remain speculative.

A clear attachment site between the nuclear membrane and the indenting droplet, eliminating the distance between the two surfaces, was visible. Here too, it remains unknown whether an exchange of lipids (e.g., sterol ester with their amphiphilic character) between nucleus and lipid droplet *via* nuclear pores will take place.

It is also important to point out a semantic inaccuracy often found in literature. The term *Lochkern* is sometimes referred to as “vacuolated” nucleus and some reports mention various forms of “vacuoles” which may be present in the nuclei of distinct cells (Bavle, 2016). From a histological point of view, “vacuoles” are enclosed cavities in eukaryotic and prokaryotic cells which serve as storage place for numerous substances including ions, nutrients and waste. As already explained above, there is convincing evidence that *Lochkerne* do not harbor vacuoles *sensu stricto*. The apparent holes in the adipocyte nuclei are the result of an unintentional loss of the lipid content elicited by chemicals used during tissue processing for light microscopy. In contrast, during tissue processing for ultrastructural analyses lipids are fixed and stabilized by osmium tetroxide. Therefore, electron-optical images never show nuclear “holes” and the preserved lipid droplets can be analyzed *in situ.* In this way, we were able to depict the gradual approach of lipid droplets towards the nucleus and their final attachment to the nuclear membrane.

In summary, the results from our morphological analysis support the notion that, from a morphological point of view, lipedema is indeed a lipomatosis. Future studies should focus on structural and genetic similarities between lipedema and lipoma pathology. Combining ultrastructural analysis with transcriptomic or molecular approaches will be required to determine whether nuclear indentation in lipedema adipocytes is associated with altered chromatin organization or transcriptional activity. This could expand the field of lipedema research and would contribute to a deeper understanding of the pathogenesis of lipedema.

### Limitations of the study

The small sample size is a major limitation of the study, and future research should include larger cohorts to confirm our findings. Information on patients’ medical history is limited and characteristics such as the duration of the disease is missing. Furthermore, additional information on the genetic background of the patients (e.g., hereditary or non-hereditary form) would be informative. The average values for BMI and age differ between the lipedema and control groups. However, this disparity was mitigated by the finding that the frequency of LK is not related to age or BMI.

## Data Availability

The original contributions presented in the study are included in the article, further inquiries can be directed to the corresponding authors.
